# Alternative Careers toward Job Market Integration: Barriers Faced by International Medical Graduates in Canada

**DOI:** 10.3390/ijerph20032311

**Published:** 2023-01-28

**Authors:** Tanvir C. Turin, Nashit Chowdhury, Deidre Lake

**Affiliations:** 1 Department of Family Medicine, Cumming School of Medicine, University of Calgary, Calgary, AB T2N 1N4, Canada; 2 Department of Community Health Sciences, Cumming School of Medicine, University of Calgary, Calgary, AB T2N 1N4, Canada; 3 Alberta International Medical Graduates Association, Calgary, AB T2E 3K8, Canada

**Keywords:** international medical graduates, alternative career pathway, career choice, barriers

## Abstract

International Medical Graduates (IMGs), who completed their medical degree and training outside Canada constitute a notable portion of the skilled migrants of the country. However, due to a long and uncertain licensure process and limited opportunities many IMGs look for alternative career pathways where they can utilize their learned skills. Alternative careers in the health and wellness sector may offer such opportunities; however, IMGs’ success in these pathways were also less evident despite their high potential. In this study, we investigated the barriers that IMGs stated to face when attempting alternative jobs in Canada. Eight focus groups with 42 IMGs in Canada were conducted. Using a thematic analysis approach, we identified that IMGs encounter these barriers in different stages of their resettlement journey in Canada, including both the pre-migration and post-migration phases. In the pre-migration phase, IMGs were not aware of the success rates of the licensing pathways and did not have sufficient information regarding potential alternative careers. In the post-migration phase, the lack of information continues to affect IMGs where IMGs exhaust their resources pursuing alternative careers without proper guidance and support. Further, IMGs struggle with taking preparation for alternative careers by obtaining further certifications and completing other prerequisites for some barriers, such as financial constraints. While looking for jobs, some IMGs perceived systemic discrimination such as non-recognition of their credentials and experience. Furthermore, the mismatch of expectations and limited growth opportunities offered by potential careers serve to disincentivize IMGs from pursuing an alternative career. Addressing the current employment inequity experienced by IMGs in Canada warrants research collaborations between organizations supporting IMGs and policymakers that target known barriers to the pursuit of alternative careers by IMGs.

## 1. Introduction

International medical graduates (IMGs) are individuals who have graduated from medical schools located outside of the countries they aim to practice as physicians [1]. In Canada, most IMGs are immigrants, refugees, or temporary migrants who immigrated to Canada after completing their medical graduation. Others are Canadian-born citizens who studied medicine abroad and came back to Canada to practice as physicians [2]. While IMGs may have had successful medical careers in the countries they grew up and/or obtained their medical degrees, they often become un- or under-employed in the country they attempt to professionally integrate because of demanding and resource-intensive licensing procedures [3,4]. Unfortunately, many IMGs symbolize the de-skilling of highly skilled migrants in developed countries [5]. A substantial portion of IMGs are unable to pursue their intended career of becoming practicing physicians in Canada because of individual-level and system-level hurdles [6,7]. While individual barriers such as financial constraints, lack of social networks, and acculturation affects their professional success [8], the key barriers are those at the system-level, particularly the limited spots in residency training that largely creates the bottleneck for IMGs to enter into medical practice. In 2020, only 22.6% of IMGs were able to get into residency training and historically, the number has been 10–18% [8,9,10]. To put this number into contrast, the same rate for Canadian medical graduates was 97.7%, and for IMGs in the US, it was 61.1% [9,11]. After assessing the process of residency recruitment in Canada, MacFarlane argued that the current assessment of criteria for IMGs is not transparent, not objective, not impartial, and not fair, restricting IMGs to a very small number of residency programs and specialties, resulting in a huge number of IMGs remaining unlicensed [10]

This low success rate pushes IMGs in Canada to seek alternative career options where they can potentially employ their education and training in medicine to a certain extent [5]. Alternative careers are the career options that IMGs may wish to pursue due to the similarities of the required knowledge, skills, job responsibilities, and rewards with their original profession, to some extent [3,4]. Nevertheless, pursuing alternative careers presents a significant challenge for IMGs as many are oblivious to their level of knowledge and skills in relation to potential alternative careers in Canada [6]. They are also less knowledgeable about how to get ready for such alternative careers, including how to find information and resources [12]. However, not much is known about the barriers to alternative careers that IMGs encounter. Thus, this exploratory study seeks to understand IMGs’ perceptions of barriers that inhibit their pursuit of alternative careers.

## 2. Theoretical Underpinnings

The immigration policies of Canada are designed to accrue human capital that welcome highly skilled migrant professionals based on their merit, education, and experience [13]. However, once they are in Canada, skilled migrants such as IMGs need to deal with multiple systems and their varied policies, which are often not in sync with Immigration, Refugees, and Citizenship Canada (IRCC). Paul and colleagues revealed that IMGs have difficulty finding a job because they must negotiate three incoherent policy systems: immigration, credential recognition and regulatory bodies, and human resources management [14]. This imposes a deskilling process on IMGs, leading them to be employed in such professions that do not use their hard-earned and extremely valuable skills and experience at all or to a minimal extent [15,16]. This illustrates a common example of systemic discrimination against immigrants [15,16]. The discrimination IMGs face when pursuing alternative careers may also be exacerbated by the interplay of colour, gender, power, law and order, immigrant status, and contemporaneous social practices [8,10]. The current inequalities in integrating IMGs into the labour market may be explained by the role that systematic prejudice plays, according to critical race theory (CRT) [17]. The failure of Canada’s credential regulatory system and human resource management to recognize IMGs’ human capital and facilitate their professional integration may be perceived as discrimination to IMGs based on their race, country of origin, education, gender, socioeconomic status, linguistic, and cultural backgrounds [18]. Previous research has shown that systemic exclusion frequently manifests as onerous restrictions placed on specific professions by regulatory bodies, which may disproportionately impede the employment of IMGs [17]. Additionally, research has indicated that IMGs from low-income regions are substantially less likely than their counterparts from higher-income ones to find their primary or alternate occupations [19]

## 3. Methods

We opted for an exploratory qualitative descriptive approach in this study as alternative career pathways for IMGs is a research area where there has been very little prior empirical work [20]. Focus group discussions (FGDs) through an online meeting platform, Zoom (Zoom Video Communications, Inc., San Jose, CA, USA), were utilized in this study as the data collection tool from the participating IMGs due to COVID-19 restrictions.

### 3.1. Recruitment

We have used the purposive sampling method in this study, which is a non-probability sampling technique where the participants are chosen by the researchers who they deem to be more relevant and appropriate for their study [21]. After our initial advertisement through posters and our community partner, Alberta International Medical Graduates Association, we were able to connect with a number of IMGs. Following a brief communication with them, we purposefully selected only those IMGs who were either already employed in alternative careers or had invested interest in pursuing one. They also shared a common background as IMGs with experiences of encountering various barriers to pursuing their careers that made the focus groups homogenous. The homogeneity was encouraged as the goal was to stimulate a deeper exploration and create an environment where the respondents were likely to feel comfortable sharing their concerns and lived experiences [22]. This sampling process ensured that we recruited only those IMGs who were proactively working towards an alternative career to learn from their lived experiences. Ethical approval was granted by the Conjoint Health Research Ethics Board (CHREB) at the University of Calgary.

### 3.2. Data Collection

Four to seven respondents participated in each focus group. The research team employed a semi-structured questionnaire that was endorsed by two citizen IMG researchers who assisted in this study. Using Zoom’s audio recording tools, each FGD was audio recorded before being transcribed verbatim. Every FGD was conducted in English and lasted about 1–1.5 h.

### 3.3. Data Analysis

To analyze the data, we used an inductive thematic analysis method [23]. We used the NVivo qualitative data analysis program (QSR International, Version 12, Melbourne) to code the verbatim transcriptions of the interviews. After coding the first three FGDs, initial codes were discussed among the research team to limit biases and identify emerging themes. The team members again met after completion of coding of data from all focus groups to finalize the codes and themes. To ensure the validity of the data multiple team members including IMGs reviewed the identified codes and themes to assess the validity of the data. The quotes and the results were also reviewed by a representative sample of the respondents.

## 4. Results

### 4.1. Respondent Demographics

We conducted a total of eight focus groups comprising of 42 participants (4–7 participants in each group). The respondents’ demographic characteristics are displayed in Table 1. The majority of respondents were female (73.8%) and were between the ages of 40 and 49 (33.3%). The majority of the respondents resided in Alberta (71.4%). Canadian citizens (47.6%) and permanent residents (47.6%) constituted the bulk of the respondents. Only two respondents (4.8%) were temporary migrants.

Family medicine/General Practice was the most frequent area of specialization for the participating IMGs prior to moving to Canada (38.1%). At the time of discussion, the majority of the respondents (59.5%) were employed either full-time (33.2%) or part-time (26.2%). However, a substantial proportion of the respondents (40.5%) were unemployed. Among employed respondents, most worked in non-regulated alternative occupations (60.0%) related to health care that do not require a licence. Approximately half of the respondents (47.6%) spent 1–3 years pursuing alternative careers.

### 4.2. Barriers to Alternative Careers for IMGs

**Figure 1:**
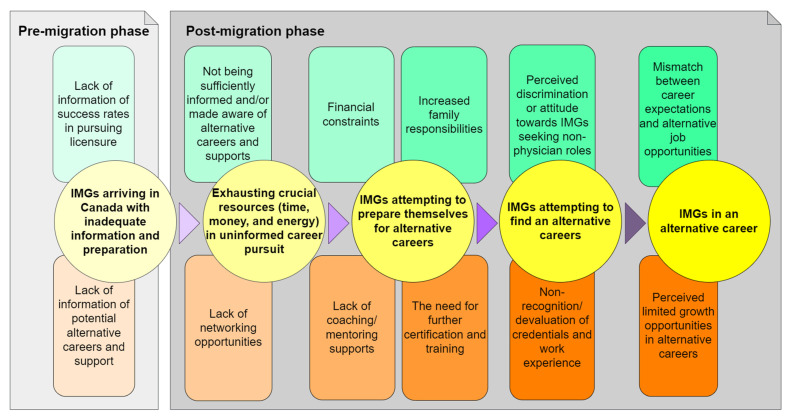
Distribution of barriers to alternative career pursuit for IMGs across their resettlement journey in Canada.

After conducting a thematic analysis of the initially identified codes, we found 12 barriers that fall under five themes. As we recognize that these barriers affect IMGs at different milestones of their migration and resettlement journey in Canada, we divided the barriers and the themes into pre-migration and post-migration phases. Figure 1 illustrates how these barriers affect IMGs in various steps of their resettlement and alternative career pursuit.

**Pre-Migration Phase**

IMGs arrive in Canada with inadequate information and preparation about different career pathways. This may include the difficulties associated with the licensing pathways to becoming practicing physicians and the associated success rates. This may also include information about potential alternative career pathways and ways to prepare and avail them.

#### 4.2.1. Theme One: IMGs Arrive in Canada with Inadequate Information and Preparation

##### Lack of Information of Success Rates in Pursuing Licensure

The participants mentioned that before coming to Canada, they did not truly understand the depth of the difficulties they had to face to become licensed physicians in Canada. They did not have sufficient information on the success rates of becoming practicing physicians in Canada.

“*So, I came to Canada. Um, before coming here, I searched or looked for like, you know, the procedures of medical licensing. I wasn’t able to, um, find a lot of information or I was not familiar with the system, so I’m not sure what to do and what not. So, I applied, uh, to public health at that time*…”

“… *Uh, but uh, I was rejected that time. So, I came in as a PR (Permanent Resident). So, when I came in, um, I, um, I even then I continued with the medical licensing exams. Uh, I gave, MCCEE (one of the licensing exams for becoming a practicing physician in Canada). Um, and then, um, things like, uh, I found that the information’s, uh, first of all, there, we don’t have a lot of information about the exams or the procedures and how to get started relocating into new countries as, as a, as a whole thing. And it’s a, it’s a tough thing*…”

“… *So yeah, I gave MCCEE and then, um, I figured that it’s going to be very hard for me. And then I got the bigger picture, uh, that. You know, there’s like only 10 or 15% gets in into the medical field or licensing*.”

(FGD 8, Male, 30–39 years, Employed, Pursuing alternative career for 1–3 years)

##### Lack of Information of Potential Alternative Careers and Support

A lack of information of potential alternative careers for IMGs before coming to Canada was mentioned by several IMGs during the discussions.

“*So, coming here, you know, you don’t really have a lot of information concerning alternative careers to go into. If you decide that, I don’t want to go with licensure and it’s now hard to know, can I do a full-time job? Can I be flexible? What’s uh, what’s is it going to [be], what am I going to earn, if I go into this career, there’s really no information out there*.”

“*You have to do your [own]. research and that might take a lot of time. And, doing your own personal research you might not actually get appropriate information. And so, all that time, you’re wasting time*.”

(FGD 3, Female, 30–39 years, Unemployed, Pursuing alternative career for 1–3 years)

**Post-Migration Phase**

IMGs endure encountering various barriers as they gradually go through different stages of resettlement and career building in Canada.

#### 4.2.2. Theme Two. Exhausting Crucial Resources in Uninformed Career Pursuits

##### Not Being Sufficiently Informed and/or Made Aware of Alternative Careers and Supports

The lack of adequate information of potential alternative careers also follows IMGs in their early days after migration. Due to insufficient information, they exhaust their limited resources (time, money, and energy) after licensing exams and/or unnecessary courses in order to pursue alternative jobs.

“*And something that struck me when I immigrate here is that I only know how to be a physician. And right now, I cannot be a physician. So, I was like, seeing like, okay, what can I do? How can I use my skills for something else? Because also like my savings, once you take your savings [from back home]. and put it into dollars [converting into Canadian money]. it changes completely [loses its value]. and the exams take time. So, I was like, I was looking for jobs and I start asking, like, what Canadians, they cannot even see from your eyes with the whole concept of like [becoming]. doctors [here], they don’t understand very well the process for us*.”

(FGD 7, Female, 30–39 years, Employed, Pursuing alternative career for 1–3 years)

“*So, I need to balance and give my priority to my kids. So, I. Just choose based on this, whatever the job, it gives me the money, but I want to fit into these criteria [job requirements], because I have already spent a lot on my licensing examination*.”

(FGD 2, Female, 40–49 years, Unemployed, Pursuing alternative career for 1–3 years)

“*You might not actually get appropriate information. And so, all that time, you’re wasting time that as the [another participant]. said, that, you know, she had to go on to [become a]. medical office assistant. She went to school to study that took a lot of time. If you speak to a lot of people and people tell you, Oh, you don’t need to go to school if you already have a medical degree. She did hospital administration too, but then I’m sure she didn’t know that. So, all those kinds, things like that. We don’t know that. Second, the [course]. you studied, you spent money then after that you find out you didn’t need to do that, or you should have done that after. So, I think information is something that’s really very important for IMGS when we come here*.”

(FGD 3, Female, 30–39 years, Unemployed, Pursuing alternative career for 1–3 years)

Another participant compared IMGs with skilled migrants from other professions and argued that IMGs do not have similar support.

“*I just want to give you an example of my husband. He’s a mechanical engineer. There were some organizations over here who are working with them and he’s, he is, uh, like, I think that he’s very much lucky that he’s an engineer and there are organizations who work with engineers, they put them on the placements and then they get a job. I think for IMGs, this is another thing that we don’t have any information on an alternative career pathway. We don’t have any agency for that one who worked collaboratively with them and then put them in some alternative career pathway. But that’s the main thing where we are missing. We do one thing, then we don’t have a growth chance over there, or we don’t get satisfied then move to another one. Then we have to spend another year [and]. money, [that affects]. our finances, our stress, our home. And then again, the whole thing starts from scratch. So, this is one thing that we are missing, we don’t have any organization that works in the alternative career, pathway, particularly for us, so that we have information to choose [from]. And then we have, we know that there is something where we can go back and ask from them*…”

(FGD 3, Female, 40–49 years, Employed, Pursuing alternative career for <1 year)

##### Lack of Networking Opportunities

Lack of networking came up as a key barrier during the discussions. IMGs indicated that since they had to compete with locals for alternative jobs and the locals had an established social network here, it was difficult for IMGs to develop a network as an immigrant and to get employed in alternative careers.

“*I figured that, you know, as you’re competing with the Canadian professionals, it’s not very easy. It requires a lot of networking, a lot of know-how*.”

(FGD 4, Male, ≥50 years, Employed, Pursuing alternative career for 1–3 years)

One IMG respondent shared their experience of the ‘informal’ need of being referred by someone to be considered for a job.

“*I have put [my resume]. on the websites. I usually go on to check [websites]. That includes AIMGA (Alberta International Medical Graduates Association), and [other]. career websites, such as UofC (University of Calgary), AHS (Alberta Health Services). I think, from the career transition program I have attended and from the mentors, what I have heard is in Calgary, the [job]. applications do not work directly in a sense. It [is]. just the networking game of getting into something. So, uh, and they had told me, if it was Toronto, it would be transparent in the sense they will take [you]. directly from your application [without the need of a referral], but that’s not how it works in Calgary. So, even though AIMGA comes up with a list of links [of potential jobs], I don’t think it’s going to be effective or [they will get]. back to [us], that’s what I feel. I’m not sure*.”

(FGD 1, Female, 40–49 years, Employed, Pursuing alternative career for 1–3 years)

It was discussed that volunteering opportunities could be useful to overcome these barriers. Through volunteer work, one may build certain connections that lead to their employment.

“*But they said that for going into the medical field as well, any field you want to go, you just do the volunteer[ing]. first. You need connections, whatever you [can get]. towards that. And you need some work experience over here. So, before that, I just went to [mumbled; a research organization], and over there I did one year of the volunteership and then they hired me over there*.”

(FGD 6, Female, 30–39 years, Unemployed, Pursuing alternative career for <1 year)

#### 4.2.3. Theme Three: IMGs Attempting to Prepare Themselves for Alternative Careers

##### Financial Constraints

For many alternative careers, IMGs might need to take certain preparations including completion of some courses, attaining certificates, volunteering, and so on. Financial constraints may limit their ability to adequately prepare for alternative careers.

“*To study something, it requires money, and I wouldn’t want to burden myself with more finance or more loans. So, I opted for this [a job that did not require further study, thus no money]*.”

(FGD 5, Female, ≥50 years, Employed, Pursuing alternative career for >5 years)

Especially, for those IMGs who had landed recently and had a family and children to take care of, it was an additional financial burden for them to afford courses required for alternative careers.

“*And secondly, again, then again, um, the financial constraints of it. Um, sometimes you are able to get OSAP [student loan for Ontario students]. because currently, I’m in Ontario. So yeah. Um, you are able to, you know, get some financial help. Um, but, if say, for example, we were talking about someone who just landed and you know, it’s a couple who have kids. Um, it’s harder for them to manage a long term, um, a long study or a long course that, uh, the, both of the spouses are enrolled in because of the finances*.”

(FGD 3, Female, ≤29 years, Unemployed, Pursuing alternative career for <1 year)

##### Increased Family Responsibilities

There was a discussion about how family responsibilities, in particular, taking care of children, may add additional stress to preparing oneself for alternative careers.

“*So, I had to give time to her [my baby]. and be flexible because I was living with my in-laws, so many things. So then, I chose, um, an online, uh, diploma program for health information management*.”

(FGD 3, Female, 30–39 years, Unemployed, Pursuing alternative career for 1–3 years)

##### Lack of Coaching/Mentoring Supports

The respondents discussed the lack of organizational or systematic support such as bridging programs for most alternative careers.

“*So, I think. That there needs to be some sort of, mention that there should be some sort of bridging [program], right? So that bridging [program]. that helps not just us in, um, providing our, uh, in providing our services, but also like helping out people who need them*.”

(FGD 3, Female, ≤29 years, Unemployed, Pursuing alternative career for <1 year)

It was also pointed out by one respondent that while there are some organizations trying to support IMGs in pursuit of alternative careers, they were not available across Canada or open to all based on funding restrictions.

“*And then again, the whole thing starts from scratch. So, this is one thing that we are missing, we don’t have any organization that works in the alternative career, pathway, particularly for us, so that we have information to choose. And then we have, we know that there is something where we can go back and ask from them*.”

(FGD 3, Female, 40–49 years, Employed, Pursuing alternative career for <1 year)

Another discussion point was that the many immigration-serving organizations might not have sufficient knowledge of alternative careers for IMGs. When an IMG went for employment support, these organizations often referred to them as low-skilled survival jobs that did not utilize IMGs’ medical knowledge and skills. Anything about how organizations such as AIMGA which understand IMGs how are useful would be beneficial to highlight following this.

“*I was not looking for anything outside though. Um, all this, uh, immigration counselling and support services, they were just pushing towards, you know, survival jobs, which I, I accept, I acknowledge, I respect all types of jobs, but [they were only pushing us to]. survival jobs [which are]. outside of health sector like Walmart, Superstore. And I was thinking if it’s a survival job, then it has to be within healthcare, nowhere else, because I cannot just imagine myself doing something else [other than healthcare]. Um, and the opportunities are, um, I can describe them as, um, maybe applying 200, 300 applications*.”

(FGD 5, Female, 40–49 years, Employed, Pursuing alternative career for 1–3 years)

##### The Need for Further Certification and Training

Respondents frequently mentioned the need for further certification and training for many alternative careers. A few IMGs accorded with the requirement and showed their will to attain further certification and training for an alternative career.

“*Um, because, uh, I knew for the fact that, uh, in Canada, you have to get a Canadian degree to get into the system. That was so like, I was very clear in my mind that I had to get a degree from here. So, then I chose the diploma. I, there were a lot of things going on in my family*.”

(FGD 3, Female, 30–39 years, Unemployed, Pursuing alternative career for 1–3 years)

However, others largely felt that this requirement will need devoted time and money from IMGs and were skeptical if that was worth it.

“*Yeah, we have to go back to the start and do university again or college again. Then, you know, that, uh, that takes a lot of resources from us and makes it difficult*.”

(FGD 1, Male, 40–49 years, Unemployed, Pursuing alternative career for <1 year)

Another respondent pointed out that even though they had a higher level of degrees and training than the requirements of a particular alternative career, they still needed to redo the entire program.

“*But I discovered that in order to do so, the most basic requirement is usually, uh, for a person to be [a nurse], a graduate of a nursing degree and be registered with, uh, the professional association for nurses [is required]. So, in order to get into, so I realized, to get into that field, I’d have to go into nursing first, despite being already a doctor and despite having an MBA specifically in health and healthcare*.”

(FGD 1, Male, 40–49 years, Unemployed, Pursuing alternative career for <1 year)

One respondent with children mentioned that it might be a waste to invest in themselves for alternative careers over their kids’ education. They would rather limit themselves to alternative jobs that do not require further training and certifications.

“*I have been looking into different possibilities, but what I find it, I have to get a kind of certification in many of the areas. And at this point of my life, I would say I would rather invest in my children’s education rather than putting a certificate for myself. So I look for whatever areas where I can find, and I put my effort there*.”

(FGD 1, Female, 40–49 years, Employed, Pursuing alternative career for 1–3 years)

It was also pointed out that for some alternative careers, there were certain prerequisites in the form of training courses, experience, or voluntary activities required that worked as important barriers. For example, one respondent thought of being an Advance Care Paramedic but to be enrolled in that program, they were required to do the Primary Care Paramedic program, which takes an additional 2–3 years. This particularly affects IMGs since they already have the required training and skills equivalent to a Primary Care Paramedic as a part of their training and experience as physicians back home.

“… *so, I was looking into it (Community Paramedic Support) and then learned to get into this program, someone has to do the primary care paramedic program and have to do again. […]. And, uh, they (IMGs) have to be a [primary care]. paramedic for two or three years, and then to be eligible for that program, which is a very long route for someone who wants to do that, especially if we’re looking again at our situations as being moms and being at home like, you know. So, these kinds of things are withholding us from proceeding into anywhere where we can fit*.”

(FGD 3, Female, ≥50 years, Employed, Pursuing alternative career for >5 years)

Another respondent mentioned that some of the prerequisites are often not clearly posted on the website. Therefore, they spent years applying for a program only to learn about the additional requirements.

“*Though, it’s not their requirements, it’s not written, but they [program advisors]. verbally say to me that you have to give us at least 80 h of volunteer experience. So, this was it totally new thing for me, because I have never volunteered before coming here to Canada. And so I was like volunteering where? Anywhere you want. So, there was a care home just beside my residents. So, I started working there*.”

(FGD 7, Female, 30–39 years, Unemployed, Pursuing alternative career for <1 year)

The respondents also discussed the English proficiency needs that were required in various courses or programs related to alternative careers, that sometimes acted as a barrier. One IMG argued that despite having proof of English proficiency in other ways such as having research publications and international work experience, the requirement of having a certain score in an English Proficiency Test is unfair.

“*But as a simple example, after 50 publications and working for WHO for more than 15 years, how [they want us]. to go for IELTS? Is it advisable? It doesn’t make sense*.”

(FGD 1, Male, ≥50 years, Unemployed, Pursuing alternative career for <1 year)

#### 4.2.4. Theme Four: IMGs Attempting to Find Alternative Careers

##### Perceived Discrimination or Attitude towards IMGs Seeking Non-Physician Roles

IMGs encounter certain barriers when applying for alternative job positions. This may include perceived discrimination towards IMGs as foreign-trained physicians.

“*So, I think, I, I’m not sure how this can, can be overcome, but I have felt that as soon as they learn somebody is, uh, is a physician, um, there is something that changes in the attitude. Um, and then it’s the voice of one versus the voice of many, of course, each of us. It’s very difficult to overcome all those barriers, especially if the barriers are system barriers*.”

(FGD 5, Female, 40–49 years, Employed, Pursuing alternative career for 1–3 years)

“*I was trying to bring up the point of a platform, where a political platform, that talks for IMGs. Uh, the other day I was watching the news Global News, and then there was an international graduate who filed human rights complaints. Again, in British Columbia whereby he was saying, IMGs are discriminated*.”

(FGD 1, Male, ≤29 years, Unemployed, Pursuing alternative career for <1 year)

Perceived discrimination might also come from a negative attitude towards immigrants in general, and such attitudes might discourage them to continue in an alternative career, as noted by a respondent.

“*They think you’re a bit desperate because you are immigrants. So, the attitudes you get are not exactly okay. Um, I know that about though was like, why all these, I can’t really contribute more*.”

(FGD 3, Female, 40–49 years, Unemployed, Pursuing alternative career for <1 year)

It was also discussed that many Canadians might want immigrants to act like Canadians instead of accepting the diversity they bring in. Discrimination arising from this viewpoint also hindered employment through alternative careers for some respondents.

“*I think when it comes to Canadians, they think, uh, you know, we should be like them and we are not like them*.”

(FGD 1, Female, 40–49 years, Employed, Pursuing alternative career for 1–3 years)

Another IMG mentioned that discrimination against IMGs could be systemic.

“*I was thinking there might be a Canada-wide platform, might be an association for all IMGs, which, uh, advocates for the rights. Because most [of the issues]. there are systemic, you know, in a sense, [systemic]. discriminations against the IMGs. That’s why they put all these hindrances to, you*…”

“… *Uh, the, the complaints and what [IMGs]. are going through is not heard. They only consider from the Canadian perspective, but not from our [IMGs’]. perspective. So, I totally admire what the association [AIMGA]. is doing*…”

“… *But I think at the political level, uh, AIMGA, or if we can make, it may be a Canada-wide association for IMGs. Yeah, and then [we can]. talk to the government, even the federal government and sit down and, you know, discuss with them what we can offer. You know, I think that [may]. help. Because, at the end of the day the policymakers are the final people, you know, Yeah*.”

(FGD 1, Male, ≤29 years, Unemployed, Pursuing alternative career for <1 year)

The respondents discussed how this negative attitude was highlighted during COVID-19, as Canadian medical students with incomplete education and zero experience were fast-tracked into doctors to support while thousands of IMGs with extensive experience as physicians were not allowed to contribute to any capacity (in non-physician positions).

“*We can talk to patients. We can help with that, but no. So, medical students are considered better equipped or maybe closer to the system than IMGs. I don’t know if it is because they are afraid of IMGs to let them into the system or if there are any other administrative tests that maybe we need to pass some type of contract with AMA, anything, but when the time comes, like it was really a crisis, a health care system crisis and there was this manpower that could really help the system and the system [did not use them]*.”

(FGD 5, Female, 40–49 years, Employed, Pursuing alternative career for 1–3 years)

##### Non-Recognition/Devaluation of Credentials and Work Experience

The responding IMGs talked about being forced to do entry-level and minimum-paying jobs due to non-recognition of their credentials earned back home.

“*You know, um, uh, most IMGs work in entry-level positions that are, um, non-regulated because that is the easiest to get into. These are, these are the minimum paying jobs, and these are the jobs that basically, um, high school graduates get into, you know, um, on now, if you look at the competitively, our problem basically is the credentials aspect*.”

(FGD 1, Male, 40–49 years, Unemployed, Pursuing alternative career for <1 year)

One respondent pointed out that with ongoing globalization, credentials from other countries should be recognized.

“*I just want to add only one thing regarding this qualification requirements. This is a global village and other universities in other countries; they are also equally competent and are producing sellable goods*.”

“*So now it should not be that you have to have the qualifications from Canada or from the USA. We hope it is, you respect other universities also*.”

(FGD 1, Male, ≥50 years, Unemployed, Pursuing alternative career for <1 year)

Despite having the required skill set and certification for a job from back home, the IMGs felt discriminated against when they were refused to work in alternative careers due to not having Canadian education, and work experience largely prevented them from getting into an alternative career.

“*You need to go to people, need to know the system and, plus, most of the people said that you must have at least Canadian education in your resume, which I, I found that at this stage of my life, I found it very difficult to pursue a, to tell you, honestly*.”

(FGD 4, Male, ≥50 years, Employed, Pursuing alternative career for 1–3 years)

“*Like I said on one side, at the lower end of that spectrum is that we don’t have on paper Canadian education and we don’t have Canadian work experience or have limited Canadian work experience*.”

(FGD 1, Male, 40–49 years, Unemployed, Pursuing alternative career for <1 year)

This situation often caused frustration among the IMGs, especially when this barred them from even being accepted for relatively low-skilled alternative careers.

“*Everybody is saying that we need some Canadian experience at the beginning. I don’t know about it. I could not understand [the need for]. that as I have more than 10 years of experience in the medical profession*.”

(FGD 6, Female, 30–39 years, Unemployed, Pursuing alternative career for <1 year)

One IMG, however, discussed probable reasons for employers wanting Canadian education and experience. They mentioned that despite having the education and experience relevant to the job responsibilities, the morals and values in Canada might be different from back home. Therefore, IMGs need some sort of acculturation training.

“*You know, you can relate to the struggles, but you definitely need to have some sort of Canadian experience because, um, things might not be exactly the way they were back home*.”

(FGD 3, Female, ≤29 years, Unemployed, Pursuing alternative career for <1 year)

Non-recognition of credentials may also come in the form of perceived overqualification of IMGs by potential employers. Many respondents reported that they were considered overqualified by potential employers and that is why they did not get the job. For instance, one respondent mentioned that when they went for a job at a care facility for an alternative job such as an assistant for a physician’s office or care aide, they were turned down as soon as the employer came to know them as an IMG.

“*I have gone to CareWest, a lot of facilities, even as they say, you, you present yourself as medical doctors and then they say you’re overqualified. They’ll not even give you a chance*.”

(FGD 4, Female, ≥50 years, Employed, Pursuing alternative career for 1–3 years)

One respondent mentioned that even employment counsellors advised them to hide their education and training as a physician. They were afraid that employers would dismiss them because of their overqualification.

“*I had an employment counsellor that advised me that I had to downplay my role as a doctor. I sort of try not to make sure that I was a doctor because it would disqualify me for the position*.”

(FGD 4, Female, 30–39 years, Unemployed, Pursuing alternative career for <1 year)

Another respondent added on by thinking from the employers’ perspective that perhaps the employers thought as they were overqualified, they would not be a good fit for this position, and they were likely seeking that position as a temporary or transitional job.

“*I mean, I’m afraid they consider them overqualified and they think that if somebody has a medical degree is a physician, if they involve them in jobs that are less than their qualifications, they don’t feel comfortable doing that. I think this is a barrier in the way of thinking and accepting IMGs and understanding that yes, this is temporary, or this is just helping them in their career transition*.”

(FGD 5, Female, 40–49 years, Employed, Pursuing alternative career for 1–3 years)

This person was also afraid that it might be a case of discrimination or unconscious bias that existed within the system in general. Employers might not understand or accept the pursuit of alternative careers as a short-term goal for IMGs or as a place to get their foot in the door or might feel guilty about hiring a physician in the role, which is also explored in the quotes that follow.

“*I’m thinking, um, it is because of unconscious bias or what I have felt is being within the system they’re afraid of and being afraid [of something]*.”

(FGD 5, Female, 40–49 years, Employed, Pursuing alternative career for 1–3 years)

Sometimes an IMG might apply to a position, often due to their lack of specific relevant experience, that might appear to the employer as an overqualified application. They could not directly apply to the higher position as they did not have experience in that field. However, the IMGs might be thinking of that position as an entry point from which they would gain relevant field experience and adding up that experience to their high level of education, they would match and advance to a higher position.

“*For example, we are advertising a position for an administrative assistant, um, to support some initiatives, which is great, maybe start from [there]. to get involved. And then the next level can be a project coordinator, in project management, with good growth opportunity. But for this [initial] position, they are considered overqualified. And that bias is they are thinking of how can we bring someone with so many years of studying to do a job when the job requirement is Grade 12 education or maybe a bit more, but not, not to the level that we have done*.”

(FGD 5, Female, 40–49 years, Employed, Pursuing alternative career for 1–3 years)

The respondents showed frustration and confusion regarding the evaluation of their qualifications. They indicated that some employers considered them underqualified by disregarding their education and training from back home and others rejected them for being overqualified. It appeared that they were underqualified and overqualified at the same time.

“*So, either we’re underqualified because we lack, we lack some certain small details in our education or we’re overqualified. So, um, even, in AHS so, uh, it’s, I’ve [sent]. out like about a thousand applications everywhere and, uh, and only get a handful of calls. So, it’s quite difficult for, for an IMG to secure, um, those kinds of opportunities*.”

(FGD 1, Male, 40–49 years, Unemployed, Pursuing alternative career for <1 year)

Some respondents pointed out that as they came to Canada after years of training and experience back home, they were generally older than the local candidates for an alternative career. They indicated that it was difficult to compete with a local candidate being an immigrant.

“… *that’s also where that the major bottleneck is because we’re competing with, with the younger, with younger candidates who, who got it right off the bat*.”

(FGD 1, Male, 40–49 years, Unemployed, Pursuing alternative career for <1 year)

#### 4.2.5. Theme Five: IMGs in An Alternative Career

##### Mismatch between Career Expectations and Alternative Job Opportunities

Another personal barrier that reverberated among the respondents was the lack of satisfaction with the alternative positions. Since it was a new career venture, sometimes some did not find satisfaction in one job and thought of moving or moving to another job. One respondent asserted that the source of satisfaction might be the low rates of payment compared to bigger workloads and responsibilities.

“*I actually did the course and actually started working. But, when I was in the work, [I realized]. um, it wasn’t, it wasn’t something I wanted to do. I realized that this is not me. This is not my idea of what I should be doing. Apart from the fact that I was on minimum wage, and, uh, they expect a lot from you*.”

(FGD 3, Female, 40–49 years, Unemployed, Pursuing alternative career for <1 year)

Job type was cited as another source of dissatisfaction. A respondent was missing patient interaction in a medical office assistant job, which motivated them to move on to healthcare aid jobs that had patient interaction to some extent.

“*I’ve been in a lot of jobs. Like, I did a healthcare aid job, I did a medical office assistant [job], and I did even teach. But then, at the end of the day, it’s still, what’s satisfying to me is being with the patient. So, I still need that connection with patients. So that’s why I think I’m enjoying this job*.”

(FGD 5, Female, 40–49 years, Employed, Pursuing alternative career for <1 year)

##### Perceived Limited Growth Opportunities in Alternative Careers

There was a discussion about the lack of growth opportunities in some alternative careers. Those IMGs who were looking for permanent transition and thereby advancing to higher positions in the alternative career fields were deterred from pursuing such careers.

“*There comes a point where there is a limitation. Like [another participant]. was mentioning what in the IVF (In Vitro Fertilization) coordinator, um, scope became an issue. So same thing, even with LPN (Licensed Practical Nurse) at some point scope becomes an issue where they require an RN (Registered Nurse). And sometimes, uh, that’s where, when you are limited in your job action, that’s not very motivating or encouraging to pick up something new. […]. And even in the public sector, there always be one point where due to the funding or financially, there is a limitation that’s set and then you kind of hit a dead end*.”

(FGD 2, Female, 40–49 years, Employed, Pursuing alternative career for 1–3 years)

## 5. Discussion

The current study aimed at understanding the perceived barriers to pursuing alternative careers for IMGs in Canada. We identified some of these barriers in this study by analyzing the facilitated discussions among the IMGs with lived experience of pursuing an alternative career. Using the thematic analysis approach, we identified that these barriers might affect different milestones of the journey of an IMG while they settle in Canada, including both the pre-migration and post-migration phases. In the pre-migration phase, the IMGs did not have sufficient information regarding the success rate of licensing pathways and potential alternative careers. The lack of information further follows in the post-migration phase as well while the IMGs exhaust their resources in uninformed pursuits of alternative careers. IMGs also encountered barriers when they attempt to prepare themselves for alternative careers, such as by completing some courses or attaining certificates. Some might originate from systemic discrimination or negative attitudes towards IMGs in non-physician careers largely affected during applying for alternative jobs. Some IMGs also reported when the growth opportunities or satisfaction in the alternative jobs do not match their expectation, they feel barred from further pursuing that alternative career.

The IMG participants in our study reported that the lack of information support results in IMGs coming to Canada with the sole career goal of becoming practicing physicians. A similar finding was reported in previous studies. Generally, after many years of endeavour, IMGs realize that obtaining a physician licensure might not be possible for them, only to find themselves adrift among career alternatives [24]. Sood A reports that IMGs only start to think about alternative careers when they realize that it is impracticable to obtain licensure for their original profession and become very confused with their career perspectives [25]. A study that compared how IMGs and Canadians who studied medicine abroad made their career decision of becoming physicians found that while about half of the Canadian citizens had explored other professions before deciding on medicine, no immigrant IMGs had explored other career options before selecting medicine [12]. This indicates how adrift IMGs might feel when they were driven to exploring alternative careers after moving to Canada. IMGs also do not have sufficient guidance to prepare for alternative jobs, to access useful resources and find mentoring and training support [6]. Newaz pointed out that once IMGs arrive in Canada, there is no follow-up or tracking by any level of the government that could be beneficial to assess the extent and depth of the issue and devise potential solutions [26].

However, some institutional supports for alternative careers for IMGs have been reported in studies such as the Career Transition Program (CTP) at the Alberta International Medical Graduates Association and the bridging program at Toronto Metropolitan University [27,28]. Though some of these supports are federally funded, they are not known to many IMGs as well as not accessible to those living in different cities or provinces, pointed out by the respondents of this study. Moreover, these initiatives do not address the disconnection between different levels of governments, regulatory bodies, and employers, and the issues of non-recognition or devaluation of credentials, and experience and unawareness, around the need and promotion of alternative careers for IMGs in policy and practice, which was echoed in other studies [6,29]. The CTP program also reported that the success of the support program was challenged due to the unawareness of the employers to whom they were referring their IMG clients for mentorship, volunteering, or job opportunities. Nevertheless, once they had learned the situation and interacted with the IMGs of the program, they wished to have more IMG candidates in a similar fashion [27]

Some of the barriers reported by our respondents were evident among skilled migrants in Canada at large and could be considered resettlement challenges [30]. Financial barriers prevent IMGs from affording any required courses or certification for an alternative career. This may be further complicated by having a family to take care of, which was also reported in studies exploring physician licensure-related barriers and a global barrier for skilled migrants [7,31]. Due to the skilled migration policy and process in Canada that favours applicants with many years of work experience (ironically which is often not recognized in the post-migration period for employment in alternative careers), most migrating IMGs come at an age where they have a family with young kids as well as elderly parents for whom they need to provide [7,32]. The intersection of this obligation to family and limited financial support increases the difficulty to take additional preparation for IMGs.

Systemic discrimination against immigrants is a commonly reported scenario in Canada [33]. However, the IMGs in our study claimed that in addition to the usual discrimination as immigrants based on racial, gender, and cultural identity, they felt discriminated against for being IMGs, especially in alternative careers. Further, IMGs are often not accepted for alternative careers for being overqualified. While the immigrants at large (60.9%) in Canada work as overqualified in various positions [34], our respondents shared their experiences of being rejected from alternative careers for being overqualified, which put them into a difficult position of being overqualified and underqualified at the same time. It also appears that many of these barriers such as non-recognition of prior education and experience, and limited organizational support, are also common barriers that IMGs face against their original professions, i.e., physician licensure pathways [7,35,36]. The non-recognition of prior educations and experience of IMGs compels them to complete expensive re-certification and re-training processes, which intersects with their already compromised socio-economic support structure and further complicates their professional integration in Canada [37]. Nevertheless, many IMGs’ personal viewpoints and expectations about alternative careers, such as perceived lack of growth opportunity within some alternative careers and a lack of satisfaction, might obstruct their professional integration through alternative careers. Elbayoumi U reported a similar notion regarding the career transition of internationally trained pharmacists [38].

### Strengths and Limitations

This study benefited from doing focus groups online since individuals could take part from their homes throughout Canada and express themselves more freely [39]. The purposive sampling method [40,41] used in this study possibly restricted the sample from representing the overall IMG population of Canada. However, as our intention was to include only those IMGs who are actively pursuing alternative careers, it might represent that of IMGs in Canada who are pursuing alternative careers. Further, as the research team is Alberta-based, the verification process resulted in the majority (71.4%) of the respondents being from Alberta. While this makes the findings of our study more relatable to Alberta, we believe the lessons from this study can still be applicable to IMGs in other provinces in Canada who are pursuing alternative careers as IMGs across Canada. Using focus groups as the data collection tool helped us to highlight those barriers that were generally faced by the IMGs as opposed to those which could be very personal. While focus groups did not allow us to delve into a certain barrier or experience of individual IMGs unlike one-on-one interviews, it allowed us to capture different accounts of the same barrier from different respondents, thus clarifying the mechanism and extent of a barrier better [42].

## 6. Conclusions

Despite being a study in a relatively less explored area, the study findings corroborate with the limited previous studies, thus enriching the literature on this crucial issue. Having a series of spirited focus group discussions with seven homogenous groups, IMGs sharing a similar experience, we were able to identify a number of barriers that affect IMGs at various stages of their resettlement journey including pre-migration and post-migration phases. While lack of information, financial, and career guidance supports for alternative career pursuit came up as key barriers, it was also pointed out that without promoting the recognition of IMGs’ education and experience from back home at local, provincial, and employment levels, and raising awareness and normalizing alternative careers for IMGs, the integration of IMGs through alternative careers may remain difficult. Further research on prioritizing the barriers to address based on the root causes, and redressing them in collaboration with organizations supporting IMGs, could be beneficial and improve employment equity for IMGs and in turn, for skilled migrants in Canada.

## Figures and Tables

**Table 1:** Characteristics of study respondents (N = 42).

Demographic Characteristic	n	%
**Sex**		
Male	11	26.2
Female	31	73.9
**Age**		
29 or younger	4	9.5
30–39	17	40.5
40–49	14	33.3
50 or over	7	16.8
**Province currently living in**		
Alberta	30	71.4
British Columbia	3	7.1
Manitoba	2	4.8
Ontario	6	14.3
Quebec	1	2.4
**Immigration status**		
Citizen	20	47.7
Permanent resident	20	47.7
Refugee	0	0.0
Temporary migrant *	2	4.8
**Country of origin**		
Armenia	1	2.4
Bangladesh	5	12.0
Canada	2	4.8
China	1	2.4
Colombia	1	2.4
Egypt	1	2.4
India	6	14.3
Iraq	1	2.4
Mexico	1	2.4
Nepal	1	2.4
Nigeria	6	14.5
Pakistan	9	21.4
Philippines	3	7.1
Somalia	1	2.4
Spain	1	2.4
Sudan	1	2.4
United Kingdom	1	2.4
**Specialty before coming to Canada**		
Emergency medicine specialist	2	4.8
Family/general physician	16	38.1
Nephrologist	1	2.4
Neurological surgeon	1	2.4
Obstetrician	3	7.1
Occupational medicine specialist	1	2.4
Ophthalmologist	2	4.8
Paediatrician	2	4.8
Radiologist	2	4.8
Surgeon	1	2.4
Other †	11	26.2
**Current work position**		
Employed (full-time)	14	33.3
Employed (part-time)	11	26.2
Unemployed	17	40.5
**Current area of work (among employed 25 respondents)**		
Health-related (regulated alternative career, i.e., requires licensure procedure, e.g., nursing, pharmacy technician, EMS tech, sonography and laboratory technician, etc.)	5	20.0
Health-related (non-regulated alternative career, i.e., does not require licensure, e.g., health educator, health administrative officer, researcher, health policy analyst, etc.)	15	60.0
Non-health-related professional job (non-medical career build-up, e.g., engineering, business, life sciences, etc.)	2	8.0
Non-health-related non-professional job (i.e., survival job, e.g., Uber/taxi driving, store jobs, business owner, etc.)	3	12.0
**Years spent preparing for alternative careers**		
Less than a year	15	35.7
1–3 years	20	47.6
4–5 years	4	9.5
More than 5 years	3	7.1

Notes: * on a study/work/visitor visa. † Other option includes: MPH, MD, FCPS, FRCS, or other post-graduate training in various specialty.

## Data Availability

The data is not publicly available due to the conservation of the privacy of the respondents.
